# Uric Acid Provides Protective Role in Red Blood Cells by Antioxidant Defense: A Hypothetical Analysis

**DOI:** 10.1155/2019/3435174

**Published:** 2019-03-27

**Authors:** Yunxiao Song, Li Tang, Jianping Han, Yanting Gao, Binghua Tang, Mingxi Shao, Wenhua Yuan, Wen Ge, Xiaofeng Huang, Tianyue Yao, Xiaobo Bian, Shengjie Li, Wenjun Cao, Haichen Zhang

**Affiliations:** ^1^Department of Clinical Laboratory, Shanghai Xuhui Central Hospital, Shanghai, China; ^2^Department of Clinical Laboratory, Eye & ENT Hospital, Shanghai Medical College, Fudan University, Shanghai, China

## Abstract

Uric acid (UA) is a major antioxidant molecule in the human blood, and it has been linked with cell longevity. However, it is unclear whether serum UA levels are associated with red blood cell (RBC) indexes. This cross-sectional study included 10,759 Chinese subjects, recruited from the Shanghai Xuhui Central Hospital from January 2014 to December 2017. The participants were categorized into gender groups and then further divided into three different subgroups according to their UA reference range as follows: low (male (UA < 0.202 mmol/l), female (UA < 0.143 mmol/l)), normal (male (0.417 mmol/l > UA ≥ 0.202 mmol/l), female (0.339 mmol/l > UA ≥ 0.143 mmol/l)), and high (male (UA ≥ 0.417 mmol/l), female (UA ≥ 0.339 mmol/l)). The associations of UA levels with RBC parameters were analyzed using 1-way ANOVA, Pearson correlations, and multivariate linear regression. The levels of mean corpuscular hemoglobin, mean corpuscular hemoglobin concentration, RBCs, and hemoglobin were lowest in the low UA group, followed by the normal UA group and high UA group (*p* < 0.001). Pearson analysis showed that there was a statistically significant correlation between UA levels with mean corpuscular hemoglobin, mean corpuscular hemoglobin concentrations, mean corpuscular volumes, RBC counts, and hemoglobin (*p* < 0.05). Multiple linear regression analysis suggested that there were statistically significant positive correlations between UA levels and RBC counts (*B* = 0.245, *p* < 0.001, 95% CI = 0.003 to 0.092), as well as UA levels and hemoglobin concentrations (*B* = 0.138, *p* < 0.001, 95% CI = 0.002 to 0.082). Furthermore, similar results were observed in both the male and female subgroups. The serum UA levels may be independently associated with RBC parameters, regardless of sex, and UA may protect RBCs owing to its antioxidant effect.

## 1. Introduction

Uric acid (UA) oxidase, an enzyme that converts UA to 5-hydroxy isourate and H_2_O_2_, was lost in hominoids during primate evolution. This loss of UA oxidase may have evolutionary advantages [1, 2], as the average level of serum UA in human is 5- to over 20-fold higher than in most other mammals [1]. UA, a naturally occurring product of purine metabolism, is a major water-soluble antioxidant molecule in human plasma with metal-chelating properties, and it has the ability to scavenge nitrogen radicals and superoxide in plasma, which helps block the generation of the strong oxidant peroxynitrite [3–5]. The level of serum UA has been shown to have a significant positive correlation with total antioxidant potential in the blood (*p* < 0.05) [6–9].

There has been increasing experimental and clinical evidence suggesting that higher plasma UA levels may protect humans from cancer, multiple sclerosis, central nervous system diseases, glaucoma, and other life-shortening disorders [10–16]. For example, Whiteman et al. [17] have shown that treatment with UA inhibits the onset of clinical disease in an acute aggressive form of allergic encephalomyelitis in mice. Moreover, recent evidence from an in vitro study has shown an intrinsic variability in plasma UA levels that might be related to the interdonor variability observed in the storage capacity of red blood cells (RBCs), and this has led to the proposal of a model for the antioxidant effect of UA during RBC storage [18]. There was also a negative correlation between the levels of heme degradation products and RBC deformability, establishing the contribution of RBC oxidative stress to impaired deformability and cellular stiffness [19].

RBCs play an important role in the transport of oxygen from the lungs to other tissues. There is growing evidence to suggest that oxidative stress plays a significant role in damaging the RBC membrane and impairing its deformability [18–20]. During blood circulation, RBCs are particularly susceptible to oxidative stress, as they are continuously exposed to high oxygen levels, both endogenously and exogenously. The influence of unneutralized reactive oxygen species (ROS) on RBCs was damage the RBC membrane, impairing the flow of RBCs through microcirculation and the delivery of oxygen to tissue [21, 22].

Although UA has been shown to play a role in RBC storage in vitro as an endogenous antioxidant, to our knowledge, the relationship between UA levels and RBCs in human health has not been studied previously. It can be hypothesized that low levels of UA are associated with increased oxidative stress and inflammation, and UA may provide protection for RBCs through its antioxidant properties. Thus, we performed this large-sample cross-sectional study to assess the association between serum UA levels and RBC indexes.

## 2. Materials and Methods

### 2.1. Subjects

The study was approved by the Ethics Committee of the Shanghai Xuhui Central Hospital (2018025), Shanghai, China, and was conducted according to the Declaration of Helsinki. Subjects were recruited from participants in yearly health screenings of the Shanghai Xuhui Central Hospital from January 2014 to December 2017 according to the inclusion criteria listed below.

Medical examinations were performed by respective physicians for all subjects at the Shanghai Xuhui Central Hospital. Those on treatment with agents affecting laboratory parameters, including patients with hematological disorders and chemotherapy treatment, were excluded. Subjects had no diabetes, hypertension, anemia, hematological system diseases, active infections, or major systemic diseases (autoimmune diseases and cancer) that could affect the levels of erythrocyte parameters. Children (age < 14) were also excluded. Based on these criteria, 7791 subjects were excluded from the study. In addition, 488 subjects were excluded because of missing information. As part of the standard of care at the Shanghai Xuhui Central Hospital, peripheral blood samples were routinely collected and tested for UA levels and complete blood counts. Clinical and demographic information were obtained from the medical data platform of the hospital.

### 2.2. Laboratory Analysis

Complete blood counts that included mean corpuscular hemoglobin (MCH), mean corpuscular hemoglobin concentration (MCHC), mean corpuscular volume (MCV), red blood cell distribution width (RDW), RBCs, and hemoglobin were measured with the Mindray BC-5500 (Shenzhen, China) automatic blood counting system. The blood samples (2 ml) were collected in ethylenediaminetetraacetic acid (EDTA) tubes. RBC parameters were measured within 0.5 hours after blood collection. Then, another 4 ml blood sample, which was obtained in the morning after the subjects had fasted for 8 hours, was used for UA and creatinine (Cr) measurements. Serum levels of UA and Cr were measured within 3 hours after blood collection by enzymatic colorimetry using a commercially available kit (Roche Diagnostics GmbH, Mannheim, Germany). Internal controls were analyzed daily over the 3-year period, with typical monthly CVs of 2%–7% and no significant changes in measured values.

### 2.3. Subgroup Analysis

The reference range of UA was 0.2023-0.4165 mmol/l for males and 0.1428-0.3392 mmol/l for females. Therefore, the subjects were categorized into gender subgroups. According to the level of serum UA, the male subjects were divided into 3 subgroups (low UA group (UA < 0.202 mmol/l), normal UA group (0.417 mmol/l ≥ UA ≥ 0.202 mmol/l), and high UA group (UA > 0.417 mmol/l)). The female subjects were also divided into 3 subgroups (low UA group (UA < 0.143 mmol/l), normal UA group (0.339 mmol/l > UA ≥ 0.143 mmol/l), and high UA group (UA > 0.339 mmol/l)).

### 2.4. Statistical Analysis

The data were analyzed using SPSS13.0 (SPSS Inc., Chicago, IL) and GraphPad Prism 6 software (La Jolla, CA). The results are presented as the mean ± standard deviation (SD). The UA/Cr ratio is reported as UA∗1000/Cr. Normality was assessed with the Kolmogorov-Smirnov test. The chi-square tests were used to compare the categorical variables among the three groups. The one-way ANOVA test was used to compare the continuous variables among the three groups. The associations between UA levels and RBC parameters were analyzed using Pearson correlation analysis. After simple linear correlation analysis, multivariate linear regression analysis was performed to confirm RBC parameters that were associated with UA levels and UA/Cr ratios. A two-sided *p* < 0.05 was considered statistically significant.

## 3. Results

### 3.1. Characteristics of the Study Subjects

A total of 10,759 subjects (6368 males, 4391 females) were studied according to the inclusion criteria. Subjects' average age was 50.17 ± 16.22 (range, 14–89) years. The general and clinical characteristics of the study participants are presented in Table 1.

### 3.2. Comparison of RBC Parameters Stratified according to the Level of UA

**Figure 1:**
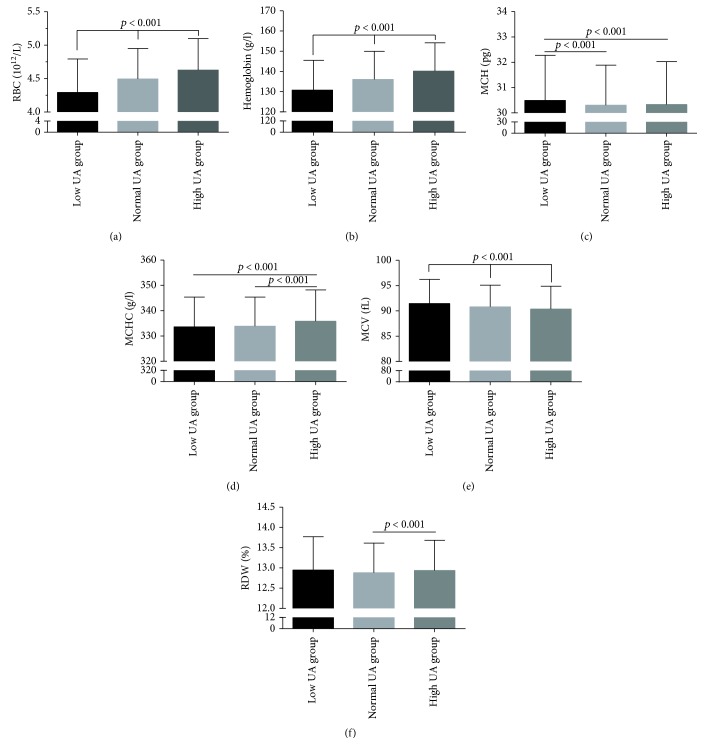
Comparison of RBC parameters in subjects, stratified according to the level of UA. Top of the box plot represents the mean and the bar of each box represents the standard deviation. RDW: red blood cell distribution width; MCH: mean corpuscular hemoglobin; MCHC: mean corpuscular hemoglobin concentration; MCV: mean corpuscular volume; RBC: red blood cell count; UA: uric acid.

**Figure 2:**
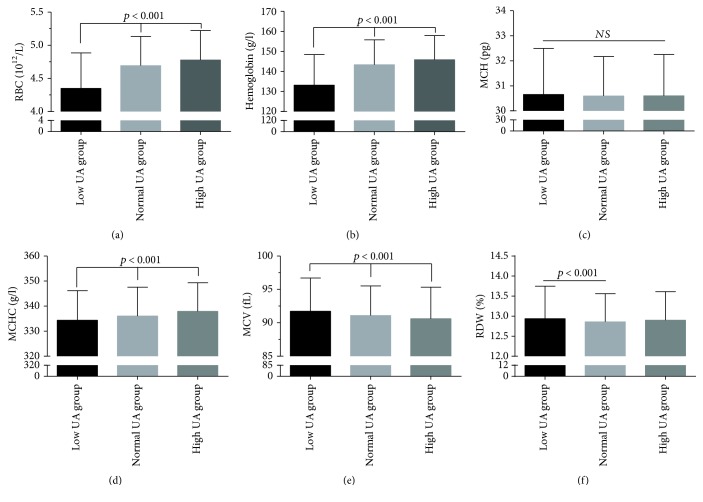
Comparison of RBC parameters in male subjects, stratified according to the level of UA. Top of the box plot represents the mean and the bar of each box represents the standard deviation. RDW: red blood cell distribution width; MCH: mean corpuscular hemoglobin; MCHC: mean corpuscular hemoglobin concentration; MCV: mean corpuscular volume; RBC: red blood cell count; UA: uric acid.

**Figure 3:**
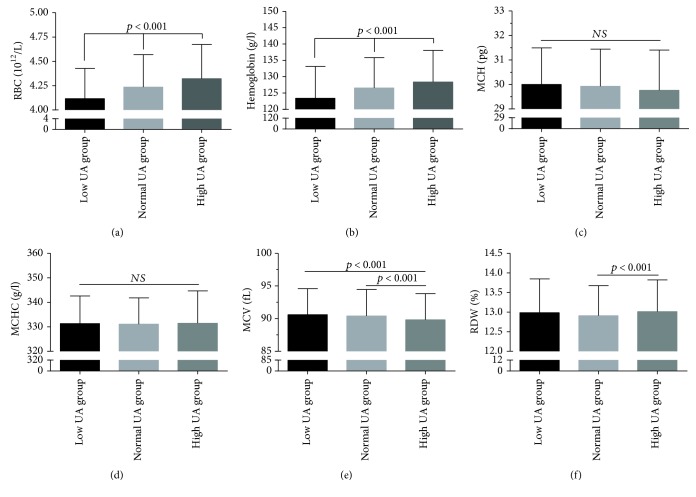
Comparison of RBC parameters in female subjects, stratified according to the level of UA. Top of the box plot represents the mean and the bar of each box represents the standard deviation. RDW: red blood cell distribution width; MCH: mean corpuscular hemoglobin; MCHC: mean corpuscular hemoglobin concentration; MCV: mean corpuscular volume; RBC: red blood cell count; UA: uric acid.

Based on the level of UA, the subjects were categorized into three subgroups: 494 subjects were classified as low UA, 8615 as normal UA, and 1650 as high UA group. A comparison of the RBC parameters in the subjects is shown in Table 2 and Figure 1. The mean levels of MCHC, RBCs, and hemoglobin were the lowest in the low UA group, followed by the normal UA group and the high UA group (*p* < 0.001). Similar results were also observed in the male subgroup (Table 3, Figure 2). The mean level of MCH was higher in the low UA group compared to both normal and high UA groups. Regarding the male subjects, MCH levels showed no statistically significant variation between the three groups (*p* = 0.815) (Figure 2 and Table 3). However, in the female subgroup, this was true only of RBC and hemoglobin levels, which were the lowest in the low UA group, followed by the normal UA group and the high UA group (*p* < 0.001); however, no correlation was found between RBC levels and MCH or MCHC levels (Table 3, Figure 3).

### 3.3. Pearson Analysis of the Association between Serum UA Levels and RBC Parameters

**Figure 4:**
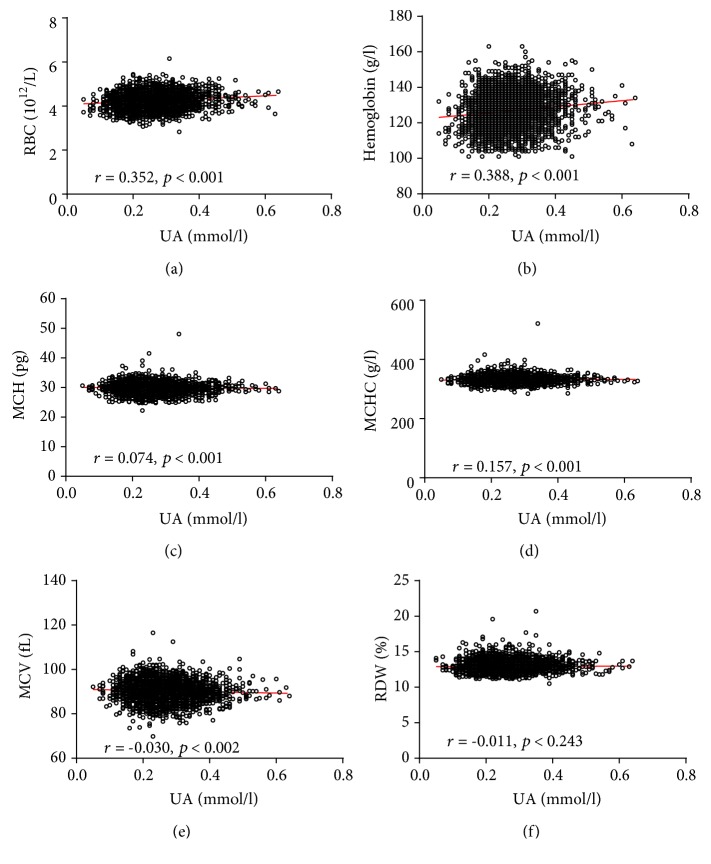
Scatterplot of individual subjects' RBC parameters versus serum UA levels. Linear regression is shown. Each data point represents one subject. RDW: red blood cell distribution width; MCH: mean corpuscular hemoglobin; MCHC: mean corpuscular hemoglobin concentration; MCV: mean corpuscular volume; RBC: red blood cell count; UA: uric acid.

**Figure 5:**
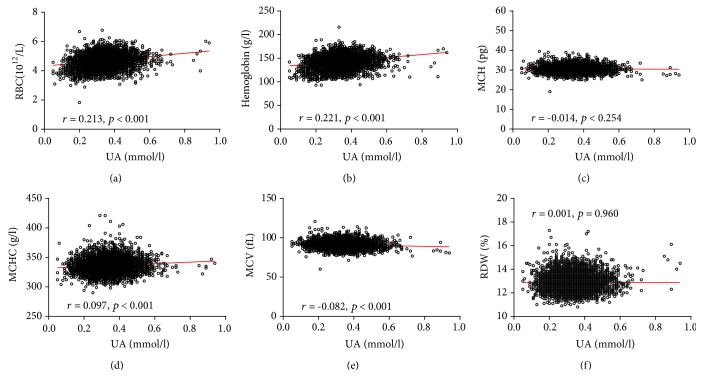
Scatterplot of individual male subject's RBC parameters versus serum UA. Linear regression is displayed. Each data point represents one subject. RDW: red blood cell distribution width; MCH: mean corpuscular hemoglobin; MCHC: mean corpuscular hemoglobin concentration; MCV: mean corpuscular volume; RBC: red blood cell count; UA: uric acid.

**Figure 6:**
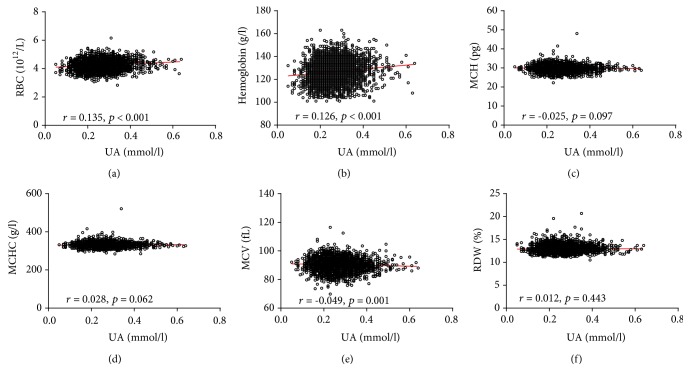
Scatterplot of individual female subject's RBC parameters versus serum UA. Linear regression is displayed. Each data point represents one subject. RDW: red blood cell distribution width; MCH: mean corpuscular hemoglobin; MCHC: mean corpuscular hemoglobin concentration; MCV: mean corpuscular volume; RBC: red blood cell count; UA: uric acid.

Pearson analyses were performed to identify the associations between serum UA levels and the UA/Cr ratio with RBC parameters. UA levels significantly correlated with MCH, MCHC, MCV, RBC, and hemoglobin levels (*p* < 0.05). Furthermore, similar results were also found between the UA/Cr ratio and RBC parameters. The specific analysis results are shown in Figure 4 and Supplementary Table 1. The association of serum UA levels and the UA/Cr ratio with RBC parameters was also analyzed in the male and female subgroups (Figures 5 and 6, Supplementary Tables 2 and 3).

### 3.4. Multiple Linear Regression Analysis of Serum UA Levels and RBC Parameters

Multiple linear regressions were used to confirm the association between UA levels with RBC parameters (Table 4). After adjustment for age, gender, and body mass index, a statistically significant positive correlation between UA levels and RBC counts was observed (*B* = 0.245, *p* < 0.001, 95% CI = 0.003 to 0.092), as well as UA levels and hemoglobin levels (*B* = 0.138, *p* < 0.001, 95% CI = 0.002 to 0.082), the UA/Cr ratio and RBC counts (*B* = 0.089, *p* < 0.001, 95% CI = 0.136 to 0.350), and the UA/Cr ratio and hemoglobin levels (*B* = 0.085, *p* < 0.001, 95% CI = 0.004 to 0.011). Furthermore, similar results were also observed in both the male and female subgroups (Table 5).

## 4. Discussion

RBCs are the most abundant cell type in the human blood; these cells contain UA but lack DNA, organelles, and protein-synthesizing machinery. Although UA contributes significantly to the radical-scavenging antioxidant capacity of plasma, the function of UA for RBCs is unclear. Several parameters were measured to see if UA could mitigate oxidative stress injury. The present study investigated the relationship between serum UA levels and RBC parameters in the blood obtained from patients. In this large-sample cross-sectional study, the level of UA was significantly associated with MCH, MCHC, MCV, RBC, and hemoglobin levels in simple linear correlation analysis. Furthermore, multivariate linear regression analyses confirmed that there were significant positive correlations between hemoglobin and UA levels as well as RBC counts and UA levels, after adjustment for potentially confounding variables. Inevitably, the serum concentrations of UA will be influenced by renal function, and we therefore used the UA/Cr ratio to reduce any possible interference in the interpretation of results caused by differences in renal function. Furthermore, similar results were observed in both male and female subgroups.

Pearson analysis was used to demonstrate that serum levels of UA are negatively correlated with MCV in both the male and female study groups. MCV is a common item included in the complete blood cell count examination package without any transformation and reflects the average volume of circulatory red blood cells. Recently, several studies reported that higher MCV was strongly associated with increased risk of death and cardiovascular disease [23, 24]. It is possible that oxidative stress may be part of the mechanism contributing to the increase of MCV with age and its association with mortality, given that oxidative stress can reduce RBC survival [25]. Hemoglobin accounts for 95 to 97% of the cytosolic protein content of RBCs and delivers oxygen to tissues throughout the body by the reversible binding of oxygen [26]. In other words, the higher the hemoglobin concentration is in RBCs, the more oxygen can be transported. In this study, a significant positive association between serum UA levels and hemoglobin was observed by Pearson analysis. Furthermore, multivariate linear regression analyses also confirmed that there was a significant positive correlation between hemoglobin and UA levels. Although limited data are available from the literature concerning the association of serum UA levels with RBCs and hemoglobin, it seems to be that higher blood UA levels may protect RBC from oxidative stress damage.

In this study, multivariate linear regression analyses demonstrated that there is a statistically significant positive correlation between UA levels and RBC counts, while Sinha et al. [6] reported that the level of serum UA was positively associated with total antioxidant concentrations. Therefore, we hypothesized that UA may function as a blood antioxidant, protecting RBCs from oxidative damage and potentially increasing RBC lifespan and function. Qasim and Mahmood [27] reported that creatine, which is also an antioxidant molecule, can prevent the induction of oxidative stress in erythrocytes caused by 2,2′-azobis(2-amidinopropane) dihydrochloride and hydrogen peroxide. Healthy individuals with low serum UA levels exhibited a higher ROS level compared to those with high serum UA levels. This has led to the proposal that higher UA levels may protect against cellular aging in normal RBCs [18]. UA was able to shield RBCs from modification and prevent build-up of carbonyl groups, as well as protect against oxidative modification of proteins.

Structural changes in echinocytes and spherical RBCs can be caused by foreign molecules (ROS, nitrogen radicals, and superoxide) in either the outer or inner monolayer of erythrocyte membranes [28]. UA was very effective in protecting the RBCs from oxidative stress damage and greatly restored RBC morphology. The protective effects of UA in RBCs can be attributed to its direct antioxidant activity, quenching free radicals and ROS. Serum UA may maintain the smooth membrane surface of RBCs, thus preventing the formation of echinocytes and spherical RBCs. Scanning electron micrographs of stored RBCs from donors showed that the prevalence of spheroechinocytosis in the blood of low UA donors was higher than that of high UA donors [18]. Decreased deformability (spheroechinocytosis) of RBCs is one of the factors that can contribute to the elimination of aged or damaged RBCs from circulation [19]. Itahana et al.'s [29] findings suggest that the p53-SLC2A9 pathway is a novel antioxidant mechanism that uses UA to maintain ROS homeostasis and prevent accumulation of ROS-associated damage. Therefore, UA was very effective in protecting RBCs from reduction in deformability and in the generation of aged or damaged RBC. Moreover, UA levels were positively correlated with RBC counts in a statistically significant fashion and negatively correlated with MCV. Hemoglobin accounts for 95 to 97% of the cytosolic protein of RBC [26], resulting in total hemoglobin concentrations that increase with increasing RBC counts in the blood. Therefore, the levels of serum UA were also positively correlated with total hemoglobin concentration, a finding that was statistically significant.

Strengths of our analysis include the relatively large sample size, the subgroup analysis including all subjects, and an analysis further adjusted for an extensive set of possible confounding variables. However, our study also has some limitations that should be considered. First, as this is a cross-sectional study, our ability to explore the exact mechanisms underlying the associations between serum UA concentration and RBC remained limited, and we did not measure endogenous and exogenous uric acid separately. Second, although children and other patients with conditions that can affect UA concentrations, including diabetes, hypertension, and anemia, were excluded from this study, several subjects with UA levels as low as 0.05 mmol/l were identified. The reason may be that it is more difficult to detect hereditary hypouricemia and xanthurenic aciduria through medical screenings; thus, such patients were not excluded from this study. Moreover, we did not consider how differences in exercise habits, smoking habits, drinking habits, and dietary preferences might affect serum UA concentrations. Third, despite the significantly low *p* values, the *r* values varied from small to moderate power in some cases. The reason may be that the diurnal variation of UA levels and day-to-day in-person variation of red blood cell indicator variables were not taken into account. Moreover, several subjects with UA levels as low as 0.05 mmol/l may influence the results of simple linear correlation. Therefore, further research was necessary in order to confirm our results.

## 5. Conclusions

In summary, our study suggested that there was a significant positive correlation between UA levels with RBC counts and total hemoglobin concentrations. To the best of our knowledge, this is the first study to report that serum UA levels may be independently associated with RBC parameters. Our findings support the hypothesis that UA plays a beneficial role in RBC longevity.

## Figures and Tables

**Table 1:** Clinical characteristics of the study participants.

	Mean ± SD	Range	Reference interval
No. of subjects	10,759	—	—
Age (years)	50.17 ± 16.22	14-89	
Gender (male/female)	6368/4391	—	—
BMI (kg/m^2^)	22.53 ± 3.10	16.51-31.30	
MCH (pg)	30.32 ± 1.61	19.0-48.1	27.0-33.0
MCHC (g/l)	334.17 ± 11.65	284-521	320-360
MCV (fl)	90.76 ± 4.35	60.1-120.7	80.0-100.0
RDW (%)	12.89 ± 0.74	10.50-20.70	9.0-17.0
RBC (10^12^/l)	4.51 ± 0.47	2.83-6.77	—
Male	4.69 ± 0.46	2.83-6.77	4-5.5
Female	4.24 ± 0.34	2.83-6.16	3.5-5.0
Hemoglobin (g/l)	136.41 ± 14.15	93-216	—
Male	143.14 ± 12.86	93-216	120-160
Female	126.66 ± 9.42	101.0-163.0	110-150
Cr (mmol/l)	72.83 ± 25.96	9-948	
Male	80.84 ± 29.33	24-948	53-115
Female	61.23 ± 13.26	9-258	50-110
UA (mmol/l)	0.306 ± 0.090	0.05-0.94	
Male	0.338 ± 0.089	0.05-0.94	0.202-0.417
Female	0.260 ± 0.069	0.05-0.64	0.143-0.339
UA/Cr ratio	4.33 ± 1.28	0.36-38.89	—
Male	4.32 ± 1.21	0.36-10.42	—
Female	4.36 ± 1.36	0.78-38.89	—

RDW: red blood cell distribution width; MCH: mean corpuscular hemoglobin; MCHC: mean corpuscular hemoglobin concentration; MCV: mean corpuscular volume; RBC: red blood cell count; Cr: creatinine; UA: uric acid; BMI: body mass index.

**Table 2:** Comparison of red blood cell parameters in study participants, stratified according to the level of UA.

	Low UA (*n* = 494)	Normal UA (*n* = 8615)	High UA (*n* = 1650)	*F* value	*p* value
Age (years)	52.69 ± 14.55	49.95 ± 15.98	50.54 ± 17.80	7.201	<0.001^a,b^
BMI (kg/m^2^)	22.40 ± 3.08	22.54 ± 3.10	22.52 ± 3.06	0.458	0.632
MCH (pg)	30.49 ± 1.78	30.30 ± 1.59	30.33 ± 1.70	3.199	0.041^a,b^
MCHC (g/l)	333.59 ± 11.77	333.89 ± 11.46	335.78 ± 12.43	18.965	<0.001^b,c^
MCV (fl)	91.45 ± 4.78	90.80 ± 4.29	90.35 ± 4.52	13.665	<0.001^a,b,c^
RDW (%)	12.95 ± 0.83	12.88 ± 0.73	12.94 ± 0.75	5.168	<0.001^c^
RBC (10^12^/l)	4.29 ± 0.50	4.49 ± 0.46	4.63 ± 0.47	114.456	<0.001^a,b,c^
Hemoglobin (g/l)	130.76 ± 14.72	136.02 ± 13.95	140.15 ± 14.02	102.371	<0.001^a,b,c^
UA (mmol/l)	0.157 ± 0.035	0.288 ± 0.062	0.446 ± 0.070	6004.230	<0.001^a,b,c^
Cr (mmol/l)	69.59 ± 16.00	70.99 ± 19.93	78.25 ± 35.64	206.204	<0.001^b,c^
UA/Cr ratio	2.45 ± 0.64	4.19 ± 0.10	5.68 ± 1.52	2106.472	<0.001^a,b,c^

RDW: red blood cell distribution width; MCH: mean corpuscular hemoglobin; MCHC: mean corpuscular hemoglobin concentration; MCV: mean corpuscular volume; RBC: red blood cell count; Cr: creatinine; UA: uric acid; BMI: body mass index. Low UA: male (UA < 0.202 mmol/l), female (UA < 0.143 mmol/l); normal UA: male (0.417 > UA ≥ 0.202 mmol/l), female (0.339 > UA ≥ 0.143 mmol/l); high UA: male (UA ≥ 0.417 mmol/l), female (UA ≥ 0.339 mmol/l). ^a^ *p* < 0.05 for the difference between low and normal (1-way ANOVA with the LSD post hoc test). ^b^ *p* < 0.05 for the difference between low and high (1-way ANOVA with the LSD post hoc test). ^c^ *p* < 0.05 for the difference between normal and high (1-way ANOVA with the LSD post hoc test).

**Table 3:** Comparison of red blood cell parameters in male and female subjects, stratified according to the level of UA.

	Low UA	Normal UA	High UA	*F* value	*p* value
Male	*n* = 374	*n* = 4879	*n* = 1115		
Age (years)	54.56 ± 14.06	50.55 ± 16.12	47.70 ± 17.45	27.711	<0.001^a,b,c^
BMI (kg/m^2^)	22.36 ± 3.15	22.49 ± 3.11	22.55 ± 3.05	0.536	0.585
MCH (pg)	30.65 ± 1.84	30.59 ± 1.57	30.60 ± 1.65	0.204	0.815
MCHC (g/l)	334.32 ± 11.84	336.05 ± 11.53	337.88 ± 11.44	17.101	<0.001^a,b,c^
MCV (fl)	91.72 ± 4.98	91.09 ± 4.44	90.61 ± 4.73	9.523	<0.001^a,b,c^
RDW (%)	12.93 ± 0.81	12.86 ± 0.70	12.90 ± 0.71	2.998	0.049^a^
RBC (10^12^/l)	4.35 ± 0.54	4.69 ± 0.44	4.78 ± 0.45	129.763	<0.001^a,b,c^
Hemoglobin (g/l)	133.13 ± 15.26	143.30 ± 12.46	145.81 ± 12.11	143.954	<0.001^a,b,c^
UA (mmol/l)	0.169 ± 0.031	0.320 ± 0.052	0.474 ± 0.060	5897.909	<0.001^a,b,c^
Cr (mmol/l)	80.43 ± 15.04	79.19 ± 20.75	81.52 ± 32.86	4.844	0.008^b^
UA/Cr ratio	2.48 ± 0.64	4.17 ± 0.95	5.57 ± 1.23	108.731	<0.001^a,b,c^
Female	*n* = 120	*n* = 3736	*n* = 535		
Age (years)	46.87 ± 14.56	49.17 ± 15.77	56.46 ± 17.05	51.531	<0.001^a,c^
BMI (kg/m^2^)	22.53 ± 2.86	22.60 ± 3.10	22.47 ± 3.08	0.390	0.677
MCH (pg)	30.00 ± 1.49	29.92 ± 1.52	29.76 ± 1.65	2.987	0.051
MCHC (g/l)	331.30 ± 11.28	331.07 ± 10.75	331.41 ± 13.25	0.235	0.791
MCV (fl)	90.59 ± 3.99	90.41 ± 4.06	89.81 ± 4.00	5.335	0.005^b,c^
RDW (%)	12.98 ± 0.87	12.91 ± 0.77	13.01 ± 0.80	4.586	0.010^c^
RBC (10^12^/l)	4.12 ± 0.31	4.24 ± 0.33	4.32 ± 0.35	24.123	<0.001^a,b,c^
Hemoglobin (g/l)	123.37 ± 9.76	126.53 ± 9.32	128.34 ± 9.71	16.390	<0.001^a,b,c^
UA (mmol/l)	0.122 ± 0.022	0.246 ± 0.045	0.389 ± 0.052	2848.194	<0.001^a,b,c^
Cr (mmol/l)	59.59 ± 12.64	60.27 ± 12.27	73.42 ± 16.49	12.774	<0.001^a,c^
UA/Cr ratio	2.33 ± 0.64	4.21 ± 1.05	5.90 ± 1.99	646.919	<0.001^a,b,c^

RDW: red blood cell distribution width; MCH: mean corpuscular hemoglobin; MCHC: mean corpuscular hemoglobin concentration; MCV: mean corpuscular volume; RBC: red blood cell count; Cr: creatinine; UA: uric acid; BMI: body mass index. Low UA: male (UA < 0.202 mmol/l), female (UA < 0.143 mmol/l); normal UA: male (0.417 > UA ≥ 0.202 mmol/l), female (0.339 > UA ≥ 0.143 mmol/l); high UA: male (UA ≥ 0.417 mmol/l), female (UA ≥ 0.339 mmol/l). ^a^ *p* < 0.05 for the difference between low and normal (1-way ANOVA with the LSD post hoc test). ^b^ *p* < 0.05 for the difference between low and high (1-way ANOVA with the LSD post hoc test). ^c^ *p* < 0.05 for the difference between normal and high (1-way ANOVA with the LSD post hoc test).

**Table 4:** Multiple linear regressions for associations between UA levels and red blood cell parameters in subjects.

	UA			UA/Cr ratio	
	*B*	*p* (95% CI)		*B*	*p* (95% CI)
Gender	0.306	<0.001 (-0.060 to -0.052)	Gender	0.107	<0.001 (0.217 to 0.336)
Age	0.032	<0.001 (0.000 to 0.001)	Age	0.092	<0.001 (0.006 to 0.009)
RBC	0.245	<0.001 (0.003 to 0.092)	RBC	0.089	<0.001 (0.136 to 0.350)
Hemoglobin	0.138	<0.001 (0.002 to 0.082)	Hemoglobin	0.085	<0.001 (0.004 to 0.011)

RBC: red blood cell count; Cr: creatinine; UA: uric acid.

**Table 5:** Multiple linear regressions for associations between UA levels and red blood cell parameters in male and female subjects.

	UA		UA/Cr ratio	
	*B*	*p* (95% CI)	*B*	*p* (95% CI)
*Male*				
Age	-0.047	<0.001 (0.000 to 0.001)	-0.156	<0.001 (-0.014 to -0.010)
RBC	0.280	0.045 (0.002 to 0.112)	0.108	<0.001 (0.165 to 0.410)
Hemoglobin	0.143	<0.001 (0.001 to 0.001)	0.093	<0.001 (0.004 to 0.013)
*Female*				
Age	0.157	<0.001 (0.001 to 0.001)	0.029	0.061 (-0.005 to 0.000)
RBC	0.094	<0.001 (0.009 to 0.029)	0.079	0.003 (0.110 to 0.524)
Hemoglobin	0.052	0.031 (0.000 to 0.001)	0.052	0.053 (0.000 to 0.015)

RBC: red blood cell; Cr: creatinine; UA: uric acid.

## Data Availability

The data used to support the findings of this study are available from the corresponding author upon request.

## Supplementary Materials

Supplementary Materials Supplementary Table 1: Pearson correlation between UA levels and red blood cell parameters in all subjects. Supplementary Table 2: Pearson correlation between UA levels and red blood cell parameters in male subjects. Supplementary Table 3: Pearson correlation between UA levels and red blood cell parameters in female subjects.

## References

[1] Wu X., Muzny D. M., Chi Lee C., Thomas Caskey C. (1992). Two independent mutational events in the loss of urate oxidase during hominoid evolution. *Journal of Molecular Evolution*.

[2] Álvarez-Lario B., Macarrón-Vicente J. (2010). Uric acid and evolution. *Rheumatology*.

[3] Glantzounis G. K., Tsimoyiannis E. C., Kappas A. M., Galaris D. A. (2005). Uric acid and oxidative stress. *Current Pharmaceutical Design*.

[4] Whiteman M., Ketsawatsakul U., Halliwell B. (2002). A reassessment of the peroxynitrite scavenging activity of uric acid. *Annals of the New York Academy of Sciences*.

[5] Ghiselli A., Serafini M., Natella F., Scaccini C. (2000). Total antioxidant capacity as a tool to assess redox status: critical view and experimental data. *Free Radical Biology & Medicine*.

[6] Sinha S., Singh S. N., Ray U. S. (2009). Total antioxidant status at high altitude in lowlanders and native highlanders: role of uric acid. *High Altitude Medicine & Biology*.

[7] Duplancic D., Kukoc-Modun L., Modun D., Radic N. (2011). Simple and rapid method for the determination of uric acid-independent antioxidant capacity. *Molecules*.

[8] Bo S., Gambino R., Durazzo M. (2008). Associations between serum uric acid and adipokines, markers of inflammation, and endothelial dysfunction. *Journal of Endocrinological Investigation*.

[9] Zabłocka-Słowińska K., Porębska I., Gołecki M. (2016). Total antioxidant status in lung cancer is associated with levels of endogenous antioxidants and disease stage rather than lifestyle factors - preliminary study. *Contemporary Oncology*.

[10] Hooper D. C., Spitsin S., Kean R. B. (1998). Uric acid, a natural scavenger of peroxynitrite, in experimental allergic encephalomyelitis and multiple sclerosis. *Proceedings of the National Academy of Sciences*.

[11] Bowman G. L., Shannon J., Frei B., Kaye J. A., Quinn J. F. (2010). Uric acid as a CNS antioxidant. *Journal of Alzheimer's Disease*.

[12] Ames B. N., Cathcart R., Schwiers E., Hochstein P. (1981). Uric acid provides an antioxidant defense in humans against oxidant- and radical-caused aging and cancer: a hypothesis. *Proceedings of the National Academy of Sciences*.

[13] Hooper D. C., Scott G. S., Zborek A. (2000). Uric acid, a peroxynitrite scavenger, inhibits CNS inflammation, blood-CNS barrier permeability changes, and tissue damage in a mouse model of multiple sclerosis. *The FASEB Journal*.

[14] Koch M., De Keyser J. (2006). Uric acid in multiple sclerosis. *Neurological Research*.

[15] Li S., Shao M., Tang B., Zhang A., Cao W., Sun X. (2017). The association between serum uric acid and glaucoma severity in primary angle closure glaucoma: a retrospective case-control study. *Oncotarget*.

[16] Tasaki E., Sakurai H., Nitao M., Matsuura K., Iuchi Y. (2017). Uric acid, an important antioxidant contributing to survival in termites. *PLoS One*.

[17] Whiteman M., Halliwell B., Darley-usmar V. (2009). Protection against peroxynitrite-dependent tyrosine nitration and *α*1-antiproteinase inactivation by ascorbic acid. A comparison with other biological antioxidants. *Free Radical Research*.

[18] Tzounakas V. L., Georgatzakou H. T., Kriebardis A. G. (2015). Uric acid variation among regular blood donors is indicative of red blood cell susceptibility to storage lesion markers: a new hypothesis tested. *Transfusion*.

[19] Mohanty J. G., Nagababu E., Rifkind J. M. (2014). Red blood cell oxidative stress impairs oxygen delivery and induces red blood cell aging. *Frontiers in Physiology*.

[20] Antonelou M. H., Kriebardis A. G., Papassideri I. S. (2010). Aging and death signalling in mature red cells: from basic science to transfusion practice. *Blood Transfusion*.

[21] Barodka V. M., Nagababu E., Mohanty J. G. (2014). New insights provided by a comparison of impaired deformability with erythrocyte oxidative stress for sickle cell disease. *Blood Cells, Molecules & Diseases*.

[22] Nagababu E., Gulyani S., Earley C. J., Cutler R. G., Mattson M. P., Rifkind J. M. (2009). Iron-deficiency anaemia enhances red blood cell oxidative stress. *Free Radical Research*.

[23] Ueda T., Kawakami R., Horii M. (2013). High mean corpuscular volume is a new indicator of prognosis in acute decompensated heart failure. *Circulation Journal*.

[24] Wu T.-H., Fann J. C.-Y., Chen S. L.-S. (2018). Gradient relationship between increased mean corpuscular volume and mortality associated with cerebral ischemic stroke and ischemic heart disease: a longitudinal study on 66,294 Taiwanese. *Scientific Reports*.

[25] Weiss G., Goodnough L. T. (2005). Anemia of chronic disease. *New England Journal of Medicine*.

[26] Rifkind J. M., Nagababu E. (2013). Hemoglobin redox reactions and red blood cell aging. *Antioxidants & Redox Signaling*.

[27] Qasim N., Mahmood R. (2015). Diminution of oxidative damage to human erythrocytes and lymphocytes by creatine: possible role of creatine in blood. *PLoS One*.

[28] Sheetz M. P., Singer S. J. (1974). Biological membranes as bilayer couples. A molecular mechanism of drug-erythrocyte interactions. *Proceedings of the National Academy of Sciences*.

[29] Itahana Y., Han R., Barbier S., Lei Z., Rozen S., Itahana K. (2015). The uric acid transporter SLC2A9 is a direct target gene of the tumor suppressor p53 contributing to antioxidant defense. *Oncogene*.

