# Supplementary material for: Uric Acid Provides Protective Role in Red Blood Cells by Antioxidant Defense: A Hypothetical Analysis
Source: Oxid Med Cell Longev. 2019 Mar 27;2019:3435174. doi: 10.1155/2019/3435174 (PMC6458867; doi:10.1155/2019/3435174)
Supplement: Supplementary Materials — Supplementary Table 1: Pearson correlation between UA levels and red blood cell parameters in all subjects. Supplementary Table 2: Pearson correlation between UA levels and red blood cell parameters in male subjects. Supplementary Table 3: Pearson correlation between UA levels and red blood cell parameters in female subjects. [file 3435174.f1.pdf]

Uric acid provides protective role in red blood cells by antioxidant defense: a hypothetical analysis

Yunxiao Song[1], Li Tang[2], Jianping Han[2], Yanting Gao[2], Binghua Tang[2], Mingxi Shao[2], Wenhua Yuan[1], Wen Ge[1], Xiaofeng Huang[1], Tianyue Yao[1], Xiaobo Bian[1], Shengjie Li[2], Wenjun Cao[2], Haichen Zhang[1*]

1. Department of Clinical Laboratory, Shanghai Xuhui Central Hospital, Shanghai, China

2.Department of Clinical Laboratory, Eye & ENT Hospital, Shanghai Medical College, Fudan University, Shanghai, China

Supplementary Table 1. Pearson correlation between UA levels and red blood cell parameters in all subjects

	UA		UA/Cr ratio	
	r	p	r	p
Age (years)	-0.013	0.173	-0.124	<0.001
BMI (Kg/m^2)	-0.007	0.466	-0.001	0.939
MCH (pg)	0.074	<0.001	0.039	<0.001
MCHC (g/l)	0.157	<0.001	0.065	<0.001
MCV (fL)	-0.030	0.002	-0.091	<0.001
RDW (%)	-0.011	0.243	0.007	0.448
RBC (10^{12}/L)	0.352	<0.001	0.133	<0.001
Hemoglobin(g/l)	0.388	<0.001	0.113	<0.001

RDW: Red blood cell distribution width; MCH: mean corpuscular hemoglobin; MCHC: mean corpuscular hemoglobin concentration; MCV: mean corpuscular volume; RBC: red blood cell count; Cr: creatinine; UA: uric acid, BMI: body mass index.

Supplementary Table 2. Pearson correlation between UA levels and red blood cell parameters in male subjects

	UA		UA/Cr ratio	
	r	p	r	p
Age (years)	-0.110	<0.001	-0.200	<0.001
BMI (Kg/m^2)	0.013	0.290	0.007	0.592
MCH (pg)	-0.014	0.254	-0.030	0.018
MCHC (g/l)	0.097	<0.001	0.103	<0.001
MCV (fL)	-0.082	<0.001	-0.102	<0.001
RDW (%)	0.001	0.960	-0.031	0.012
RBC (10^{12}/L)	0.213	<0.001	0.187	<0.001
Hemoglobin(g/l)	0.221	<0.001	0.185	<0.001

RDW: Red blood cell distribution width; MCH: mean corpuscular hemoglobin; MCHC: mean corpuscular hemoglobin concentration; MCV: mean corpuscular volume; RBC: red blood cell count; Cr: creatinine; UA: uric acid, BMI: body mass index.

Supplementary Table 3. Pearson correlation between UA levels and red blood cell parameters in female subjects

	UA		UA/Cr ratio	
	r	p	r	p
Age (years)	0.154	<0.001	-0.024	0.113
BMI (Kg/m^2)	-0.028	0.064	-0.011	0.467
MCH (pg)	-0.025	0.097	-0.046	0.002
MCHC (g/l)	0.028	0.062	0.028	0.063
MCV (fL)	-0.049	0.001	-0.074	<0.001
RDW (%)	0.012	0.443	0.052	0.001
RBC (10^{12}/L)	0.135	<0.001	0.122	<0.001
Hemoglobin(g/l)	0.126	<0.001	0.096	<0.001

RDW: Red blood cell distribution width; MCH: mean corpuscular hemoglobin; MCHC: mean corpuscular hemoglobin concentration; MCV: mean corpuscular volume; RBC: red blood cell count; Cr: creatinine; UA: uric acid, BMI: body mass index.
